# Structure and composition of supported lipid bilayers: a comparison between solvent-assisted lipid bilayer and vesicle fusion methods

**DOI:** 10.1107/S1600576726000312

**Published:** 2026-02-27

**Authors:** Birgit Felderer, Claire Buchanan, Shuhong Wang, Hsin-Hui Shen, Andrew Nelson, Stephen C. L. Hall, Marité Cárdenas

**Affiliations:** a Biofisika Institute, Leioa48940, Spain; b Fundacion Biofisica Bizkaia Leioa48940 Spain; c Monash University Clayton Melbourne Victoria3800 Australia; d Australian Nuclear Science and Technology Organisation (ANSTO) Lucas Heights2234 Australia; e ISIS Pulsed Neutron and Muon Source, Didcot, OxfordshireOX11 0QX, United Kingdom; f Department of Biomedical Science Malmö University Malmö 20506 Sweden; g Ikerbasque, Basque Foundation for Science, Bilbao 48013, Spain; Lund University, Sweden

**Keywords:** neutron reflection, supported lipid bilayers, solvent-assisted lipid bilayer formation, vesicle fusion, melittin

## Abstract

Supported lipid bilayers (SLBs) are crucial model membrane platforms to study the structure and dynamics of cellular membranes. Vesicle fusion (VF) is one of the most widely used approaches to forming SLBs, though it suffers from compositional limitations and substrate compatibility constraints. The solvent-assisted lipid bilayer (SALB) technique enables the possibility of forming SLBs using a wider range of membrane compositions and substrate platforms through organic-solvent-mediated bilayer assembly, yet questions remain regarding structural equivalence and potential organic solvent incorporation effects. Using neutron reflectometry (NR), we systematically compare the structure and composition of phosphatidylcholine-based SLBs formed by either VF or SALB methodologies. SALB conditions were optimized for NR solid/liquid cells, and structural characterization revealed comparable bilayer architectures between the two formation methods, although some changes in the lipid acyl chain thickness were observed. SALBs showed up to 99.2 ± 0.9% surface coverage using ultrapure water for solvent exchange, but the reproducibility of the method was poor. Enhanced-contrast NR using either deuterated lipids or solvents allowed for the quantitative detection of residual organic solvent incorporation of the SALBs, which was up to 3.3 ± 0.9 vol.% in the tail regions. Making use of 1 m*M* CaCl_2_ during solvent exchange substantially improved SALB reproducibility, reducing coverage variability from 21–30 to 2  vol.%. Validation studies using the antimicrobial peptide melittin demonstrated that membrane–peptide interactions proceeded according to established mechanisms, with peptide incorporation of 18 vol.% for the low-coverage (69.7 ± 0.8%) SALB. The quantified solvent incorporation levels and small changes in acyl chain layer thickness in the SALBs must be considered when interpreting protein–membrane interaction studies, which suggests that validation of the SALB methodology for membrane research applications requires assessment on a case by case basis.

## Abstract 2

Neutron reflectometry is used to compare supported lipid bilayers formed by vesicle fusion and solvent-assisted lipid bilayer (SALB) methods, revealing structurally comparable architectures with minor differences in acyl chain thickness and low residual solvent incorporation. The study establishes the structural validity and limitations of the SALB approach, emphasizing the need for case-specific validation in membrane interaction studies.

## 1. Introduction

Cellular membranes represent fundamental biological structures, presenting intricate 6 nm architectures that incorporate diverse lipid compositions and embedded proteins, and they mediate virtually all cellular functions (Clifton *et al.*, 2020 ▸). The great variety of cellular functions implies that there are no two types of cellular membrane that are exactly equivalent in terms of composition and structure. Understanding the relationship between cellular membrane structure and dynamics requires the development and use of simplified model systems that preserve essential membrane characteristics while enabling controlled experimental investigation. In this regard, supported lipid bilayers (SLBs) have emerged as essential membrane research platforms, consisting of planar lipid bilayer films deposited onto solid supports. This configuration enables the study of SLBs using diverse surface-sensitive analytical techniques (Hardy *et al.*, 2013 ▸; Ferhan *et al.*, 2019 ▸; Clifton *et al.*, 2020 ▸) to follow not only their intrinsic properties (rigidity, order, structure) but also how they behave upon being challenged by environmental factors such as the presence of nanoparticles (Rascol *et al.*, 2016 ▸), drugs (Jackman & Cho, 2012 ▸), peptides (Lösche, 2002 ▸) and antimicrobial compounds (Gidalevitz *et al.*, 2003 ▸). The use of SLBs is vast in the literature given that cellular membranes serve as targets for approximately 60% of pharmaceutical compounds (Overington *et al.*, 2006 ▸).

**Figure 1:**
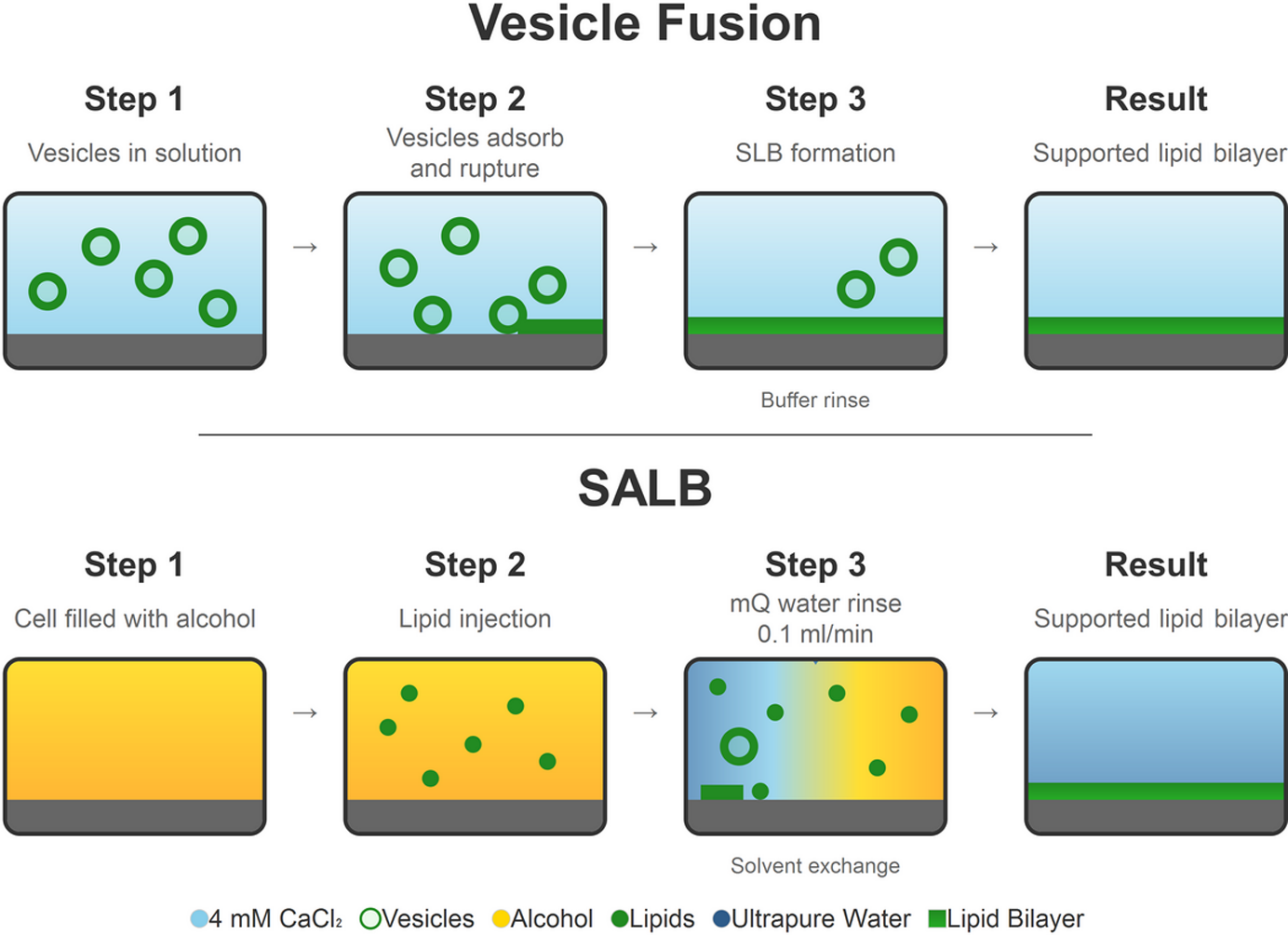
Schematic comparison of the VF and SALB formation methodologies used in this study. VF employs pre-formed vesicles in solution, their adsorption and deformation leading to vesicle rupture and the final bilayer spreading. SALB utilizes three-step solvent exchange: substrate conditioning with organic solvent, lipid injection in organic solvent, and a controlled water rinse/1 m*M* CaCl_2_ (0.1 ml min^−1^), driving sequential phase transitions to form supported lipid bilayers.

Current SLB fabrication methodologies centre primarily on vesicle fusion (VF) and Langmuir–Blodgett/Langmuir–Schäfer (LB/LS) deposition approaches (Clifton *et al.*, 2020 ▸; Hardy *et al.*, 2013 ▸; Tabaei, Choi *et al.*, 2014 ▸) (Fig. 1 ▸). Initial vesicle–substrate interactions depend primarily on the balance between attractive forces (van der Waals interactions, electro­static attraction) and repulsive barriers (electrostatic repulsion, hydration forces, steric hindrance) (Jackman *et al.*, 2014 ▸; Cho *et al.*, 2011 ▸). SLB formation can occur either through vesicle accumulation to a critical surface density followed by rupture or through direct vesicle rupture upon surface contact, depending on buffer composition (Boudard *et al.*, 2006 ▸). In general, VF success critically depends on the optimization of multiple experimental conditions (Hardy *et al.*, 2013 ▸; Jackman *et al.*, 2014 ▸). Solution pH alters the electrostatic interactions between vesicles and substrates, while temperature influences lipid phase behaviour and thermally activated rupture processes (Hardy *et al.*, 2013 ▸; Reimhult *et al.*, 2002 ▸). Ionic strength modifications control fusion pathways and kinetics (Boudard *et al.*, 2006 ▸; Jackman *et al.*, 2013 ▸). Divalent cations, particularly Ca^2+^ and Mg^2+^, enhance vesicle–substrate interactions through charge bridging mechanisms, with typical concentrations ranging from 25 µ*M* to 2–3 m*M* CaCl_2_ depending on lipid composition complexity (Hardy *et al.*, 2013 ▸; Lind *et al.*, 2019 ▸). Osmotic pressure effects control membrane tension and rupture propensity (Jackman *et al.*, 2013 ▸). Osmotic stress protocols involving vesicle exposure to elevated salt concentrations followed by dilution with deionized water can enhance bilayer formation for more complex resistant compositions, but also increase optimization complexity (Hardy *et al.*, 2013 ▸).

Despite its widespread application, VF has significant limitations that restrict accessibility to biologically relevant membrane compositions and substrate platforms (Hardy *et al.*, 2013 ▸; Jackman & Cho, 2020 ▸). Higher contents of negatively charged lipids create substantial challenges in VF through electrostatic repulsion with commonly employed silica- or glass-based substrates (Hardy *et al.*, 2013 ▸; Lind *et al.*, 2019 ▸). Furthermore, the optimization strategies require careful protocol development and may not translate across different lipid systems (Merz *et al.*, 2008 ▸). Substrate compatibility is another restriction for successful SLB formation via VF which occurs mainly for hydro­philic surfaces (silicon dioxide, borosilicate glass, mica) (Clifton *et al.*, 2020 ▸; Hardy *et al.*, 2013 ▸). Depositing lipids with a packing parameter (*P*) that deviates from unity is an additional challenge. *P* relates to the shape of the molecule by taking into consideration the headgroup area in reference to the volume of the chain multiplied by the chain layer thickness. Lipids with *P* values that deviate strongly from 1 tend to form non-planar liquid crystalline phases and are therefore difficult to deposit via VF on a flat surface (Israelachvili *et al.*, 1976 ▸). Finally, VF mostly leads to the formation of symmetric SLBs, though cases of spontaneous leaflet asymmetry exist in the literature (Wacklin & Thomas, 2007 ▸; Waldie *et al.*, 2018 ▸; Stanglmaier *et al.*, 2012 ▸; Åkesson *et al.*, 2012 ▸). These constraints motivate alternative SLB formation approaches suited to a wider variety of compositions, while maintaining the fundamental advantages of supported membrane platforms.

The LB/LS methodology offers enhanced control over bilayer asymmetry through sequential monolayer deposition, allowing independent control of each leaflet composition (Kurniawan *et al.*, 2018 ▸), but constitutes a time-consuming procedure requiring higher technical skills and normally a modified Langmuir trough, with a well, and the associated dipping/sample levelling apparatus (Lind *et al.*, 2019 ▸; Crane *et al.*, 2005 ▸; Hughes *et al.*, 2019 ▸).

The solvent-assisted lipid bilayer (SALB) method has been proposed to offer a versatile alternative to both VF and LB/LS protocols that expands the accessible membrane compositions and substrate platforms (Ferhan *et al.*, 2019 ▸). The SALB methodology builds upon reverse-phase evaporation techniques originally developed for liposome preparation (Tabaei, Choi *et al.*, 2014 ▸; Watkins *et al.*, 2014 ▸). It involves three sequential steps: (i) dissolution of lipids in water-miscible organic solvents, (ii) exposure to target substrates and (iii) progressive solvent replacement with an aqueous buffer to trigger phase transitions that culminate in bilayer formation (Ferhan *et al.*, 2019 ▸; Tabaei, Choi *et al.*, 2014 ▸) (Fig. 1 ▸). SALB formation proceeds through well characterized phase transitions driven by increasing water content (Tabaei, Choi *et al.*, 2014 ▸; Watkins *et al.*, 2014 ▸). Initially, the lipids exist predominantly as inverted micelles or individual molecules in organic solvent. Progressive water addition destabilizes these structures, promoting conversion to monomeric forms, followed by assembly into conventional micelles, and ultimately the formation of vesicle-like structures and the final bilayer (Watkins *et al.*, 2014 ▸). Phenomenological modelling established that micelles represent the primary adsorbing species responsible for bilayer assembly, with the formation kinetics limited by the diffusion of micelles through the solution rather than a surface-binding reaction (Wood *et al.*, 2021 ▸). This mechanistic understanding demonstrates that bilayer formation occurs at the same point where the micelle-to-vesicle transition is reached in the bulk solution (Wood *et al.*, 2021 ▸; Hohner *et al.*, 2010 ▸).

The SALB method has been shown to form SLBs successfully on a range of substrates including silicon dioxide (Tabaei, Choi *et al.*, 2014 ▸; Gillissen *et al.*, 2016 ▸; Hohner *et al.*, 2010 ▸; Tabaei, Jackman, Liedberg *et al.*, 2014 ▸; Tabaei, Jackman, Kim, Zhdanov & Cho, 2015 ▸), gold (Ferhan *et al.*, 2019 ▸; Tabaei, Choi *et al.*, 2014 ▸; Tabaei, Vafaei & Cho, 2015 ▸), graphene (Chin *et al.*, 2019 ▸), titanium oxide (Gillissen *et al.*, 2016 ▸), aluminium oxide (Tabaei, Jackman, Liedberg *et al.*, 2014 ▸; Betlem *et al.*, 2020 ▸) and nanoporous gold substrates (Tabaei, Ng *et al.*, 2016 ▸). SALB successfully forms SLBs over a wide range of compositions: high cholesterol fractions up to approximately 60 mol% (Tabaei, Jackman, Liedberg *et al.*, 2014 ▸; Kawakami *et al.*, 2017 ▸; Tabaei, Jackman, Kim *et al.*, 2014 ▸) and charged lipid compositions, including monovalent synthetic cationic and natural anionic lipids such as phosphatidylglycerol (PG) and phosphatidylserine (Tabaei, Jackman, Liedberg *et al.*, 2014 ▸) and multivalent anionic phospho­inositides (Kawakami *et al.*, 2017 ▸), are possible. It is also a simple technique with limited numbers of optimization parameters (Ferhan *et al.*, 2019 ▸). Amongst these the selection of the organic solvent critically influences SALB formation efficiency and bilayer quality (Ferhan *et al.*, 2019 ▸; Tabaei, Choi *et al.*, 2014 ▸). In a comparative study based on quartz crystal microbalance with dissipation (QCM-D) and fluorescence microscopy using 1,2-dioleoyl-*sn*-*glycero*-3-phos­pho­choline (DOPC) as a model membrane for mammalian cellular membranes, propan-2-ol (IPA) was identified as the optimal solvent out of five organic solvents, yielding the highest quality SLB formation in terms of coverage (Tabaei, Choi *et al.*, 2014 ▸).

Besides the choice of solvent, successful SLB formation has been shown (using QCM-D and atomic force microscopy) to depend on lipid concentration (typically 0.5 mg ml^−1^) (Hohner *et al.*, 2010 ▸; Tabaei, Jackman, Liedberg *et al.*, 2014 ▸; Chin *et al.*, 2019 ▸; Betlem *et al.*, 2020 ▸), using moderate flow rates of typically 50–100 µl min^−1^ in QCM-D, with empirical optimization required for each substrate–lipid combination (Ferhan *et al.*, 2019 ▸; Tabaei, Choi *et al.*, 2014 ▸). The collected data suggest structural properties consistent between SALBs and VF-produced SLBs (Ferhan *et al.*, 2019 ▸; Tabaei, Choi *et al.*, 2014 ▸), in terms of adsorbed mass or coverage and lateral lipid diffusion (Tabaei, Choi *et al.*, 2014 ▸). Finally, early mass spectrometry studies suggested minimal residual organic solvent incorporation in SALBs (Tabaei, Choi *et al.*, 2014 ▸).

In this work, we aim to address three fundamental questions regarding SALB methodology using neutron reflectometry (NR), as this technique excels in determining SLB composition and structure in the surface normal direction down to a resolution of a few ångströms (Clifton *et al.*, 2020 ▸). First, we evaluate the structure of SALBs and VF-produced SLBs using two widely used phospho­lipids, namely 1-palmitoyl-2-oleoyl-*sn*-*glycero*-3-phospho­choline (POPC) and 1,2-dimyristoyl-*sn*-*glycero*-3-phosphatidylcholine (DMPC), as simple models for mammalian membranes to verify whether their membrane architectures are consistent and comparable despite the method of preparation. We present a systematic optimization of the protocol, including the choice of solvent, the lipid concentration and the addition of 1 m*M* CaCl_2_ in SALB protocols to improve the method’s reproducibility. Second, we quantify any organic solvent incorporation within SALBs through enhanced contrast NR analysis using deuterated propan-2-ol, providing the first quantitative assessment of residual alcohol distribution within both head and tail bilayer regions. Third and last, we examine how potential solvent incorporation affects membrane–peptide interactions by comparing the binding mechanisms between the well studied antimicrobial peptide melittin and SLBs prepared by VF and SALB approaches. Melittin is a 26 amino acid antimicrobial peptide from bee venom (*Apis mellifera*) that carries a +6 charge at physiological pH and adopts an alpha-helical structure in solution, and its interactions with SLBs of various composition have been characterized extensively. Thus, melittin is an ideal candidate as a model for antimicrobial peptide interactions with membranes (Clifton *et al.*, 2020 ▸; Tabaei, Jackman, Kim *et al.*, 2014 ▸; Tabaei, Guo *et al.*, 2016 ▸). Our results establish the fundamental parameters governing the applicability of the SALB methodology for membrane research applications and provide structural information and evaluation of the remaining solvent content for the first time.

## 2. Methods

### 2.1. Materials

Hydrogenated (h-) POPC, hydrogenated (h-) DMPC and deuterated (d-) DMPC were obtained from Avanti Polar Lipids (Alabaster, Alabama, USA) and used without further purification. Deuterated lipids were selected to provide enhanced contrast for NR measurements and enable selective detection of hydrogenated (h-) IPA. IPA (in both hydrogenated and deuterated forms) and ethanol (EtOH) of analytical grade were purchased from Sigma–Aldrich and used for SALB preparation. Melittin was purchased from Covalab, France. Calcium chloride (Merck) solutions were prepared in ultrapure water (resistivity > 18 MΩ cm) for bilayer deposition. For VF experiments, 2 m*M* CaCl_2_ was used to facilitate vesicle attachment and rupture, following established protocols for SLB formation (Clifton *et al.*, 2020 ▸). For enhanced SALB formation, 1 m*M* CaCl_2_ was incorporated in the exchange buffer.

For contrast variation in NR measurements, Tris–HCl buffer (50 m*M*, pH 7.4) prepared in either H_2_O or D_2_O was used for SLBs prior to and after melittin addition. Tris–HCl tablets were purchased from Merck and used as received. Silicon wafers with native oxide surfaces polished to less than 4 Å roughness were used as model substrates for NR measurements. Substrates were cleaned using standard protocols involving organic solvent rinses and plasma treatment to ensure reproducible surface conditions.

### 2.2. Sample preparation

Small unilamellar vesicles were prepared for VF (Hardy *et al.*, 2013 ▸; Åkesson *et al.*, 2012 ▸). Lipid stocks were prepared in chloro­form, and aliquots corresponding to 0.7 mg were transferred to a clean glass vial, dried under a nitro­gen stream and vacuum desiccated overnight to remove residual solvent. The dried lipid films were rehydrated in ultrapure water, vortexed and tip-sonicated (20 min, 5 s on/off, 40% amplitude) on ice to achieve uniform vesicle size distributions (Åkesson *et al.*, 2012 ▸), and then diluted with ultrapure water to a final volume of 3.5 ml and mixed in equal volumes with a CaCl_2_ solution to give an end concentration of 0.1 mg ml^−1^ lipid and 2 m*M* CaCl_2_. This mixture was injected onto the cell, incubated for 10 min and then washed with ultrapure water to remove CaCl_2._

For SALB, the solid–liquid cell was filled manually with 10–20 ml of organic solvent, with subsequent manual injection of a 1.5 ml lipid solution in the same organic solvent. After 10 min of incubation, a gradual exchange of the solvent using 7 ml of either ultrapure water (h-POPC, d-DMPC) or 0.1 m*M* CaCl_2_ (h-DMPC) solution was initiated via a high-performance liquid chromatography pump at a flow rate of 0.1 ml min^−1^ to form an aqueous environment for SLB formation and to ensure complete removal of excess lipid material and residual organic solvent from the bulk solution (Ferhan *et al.*, 2019 ▸; Tabaei, Choi *et al.*, 2014 ▸; Hohner *et al.*, 2010 ▸) (the volume of liquid within the solid–liquid flow cell is slightly less than 1.5 ml). Depositions at 25°C were made for all POPC SALBs, as well as the IPA-based SALBs (0.5, 0.75, 1 mg ml^−1^) and EtOH-based SALBs (0.75 mg ml^−1^ in duplicate, 1 mg ml^−1^). The d-DMPC-based VF was performed at 37°C. One 0.75 mg ml^−1^ IPA-based SALB was formed at 37°C and another at 25°C. Challenging the EtOH-based SALB with melittin was performed at 25°C. Two h-DMPC SLBs were formed at room temperature (23°C).

### 2.3. Neutron reflectometry

The neutron reflectometry measurements used a horizontal sample geometry, on either Figaro (Campbell *et al.*, 2011 ▸) at the Institut Laue–Langevin (ILL), Offspec (Webster *et al.*, 2011 ▸) at ISIS Neutron and Muon source, or Platypus (James *et al.*, 2011 ▸, 2006 ▸; Saerbeck *et al.*, 2012 ▸) at the Australian Nuclear Science and Technology Organisation (ANSTO), with the neutron beams incident on the silicon–water interfaces through the silicon substrate. This geometry enables the investigation of buried solid–liquid interfaces under native hydration conditions (Clifton *et al.*, 2020 ▸).

Multiple isotopic contrasts were employed to enhance structural resolution and enable compositional analysis (Clifton *et al.*, 2020 ▸). Standard measurements included H_2_O and D_2_O contrasts (water measurements for the SiO_2_ surface and buffer measurements for the SLB). For samples containing deuterated lipids, silicon-matched water (SMW, 38 vol.% D_2_O) contrasts were performed to highlight different components of the SLB structure. For the detection of organic solvent incorporation, combinations of either d-DMPC/h-IPA or h-DMPC/d-IPA were used. NR data were collected over a *Q* range spanning approximately 0.02–0.33 Å^−1^ at d*Q*/*Q* resolutions of 7, 4 and 5% at ILL, ISIS and ANSTO, respectively.

For melittin interaction studies, 5 µ*M* peptide solutions (Clifton *et al.*, 2020 ▸) were introduced via manual injection, first 3 ml in D_2_O buffer (the cell was already in D_2_O contrast) and then a sequential additional 9 ml for SMW and H_2_O buffer (solvent contrast exchange) to perform prior characterization for each contrast change. This concentration was selected on the basis of literature reports demonstrating clear membrane interactions without complete bilayer solubilization (Clifton *et al.*, 2020 ▸; Pan & Khadka, 2016 ▸; Wessman *et al.*, 2008 ▸).

### 2.4. Data analysis and modelling

All SLBs were fitted to a symmetric head–tail–head model using *RefnX* (Nelson & Prescott, 2019 ▸), and the mean mol­ecular area (MMA) was allowed to vary across these layers to reflect better the variations within the structure of the SLB (head *versus* tail regions). This means that thicknesses and coverages were kept unlinked without any molecular constraint (within reasonable bounds). This is the preferred approach, since any presence of alcohol within the bilayer or low coverage effects could affect the SLB structure. In particular, SLBs with lower coverage typically present a greater variability in MMA due to the local deformation of the SLB at the edges of each patch needed to minimize the exposure of the hydro­phobic core to the aqueous bulk solution (Jiang *et al.*, 2004 ▸).

The scattering length densities (SLDs) of the lipids and organic solvent were kept constant. This approach treats the bilayer as a series of stratified layers, each characterized by a thickness, SLD and interfacial roughness. Parameters were constrained, instead, on the basis of physical expectations for the lipid bilayer structure in terms of layer thicknesses, and using the lipid volumes to calculate MMA values across heads and tails.

Parameter estimation was performed using Markov chain Monte Carlo (MCMC) sampling with an affine-invariant ensemble sampler (200 walkers). After differential evolution initialization, the chains were sampled for 1000 burn-in steps (discarded) followed by 8000 production steps with a thinning factor of 400 to determine parameter distributions and uncertainties. Reported uncertainties represent one standard deviation (68% credible intervals) of the posterior parameter Gaussian distributions. To detect and quantify organic solvent incorporation within SALB bilayers, the *RefnX* class *MixedSlab* was used (Nelson & Prescott, 2019 ▸). This method allows independent variation of lipid and alcohol volume fractions within each bilayer region, providing sensitivity to compositional variations that would be undetectable using conventional homogeneous layer models. Co-refinement approaches were employed where all two (clean surface and SLB) or three datasets (clean surface, SLB and SLB + melittin) were fitted simultaneously with shared structural parameters for solvent/water layer, roughness, head thickness and volume fraction, varying tail thickness and volume fraction to account for the incorporation of melittin, using the *MixedSlab* approach. Asymmetric models were tested but did not significantly improve the fit quality or demonstrate significant differences across the structural parameters found through the symmetric model reported here. SLD values for each layer, and head and acyl chain volumes, are given in Table S1 in the supporting information, while fitting bounds are given in Table S3.

For VF bilayers, four replicates were prepared for d-DMPC samples to establish baseline variability, demonstrating excellent reproducibility. For SALBs, two replicates were prepared for each condition, representing typical sample sizes achievable with NR studies given beamtime constraints. All sample preparations were consistently conducted by two of the co-authors using standardized protocols. Substrate preparation, cleaning procedures and sample injection were kept constant across all experiments to ensure comparability between VF and SALB methods.

## 3. Results and discussion

### 3.1. Lipid concentration optimization

**Figure 2:**
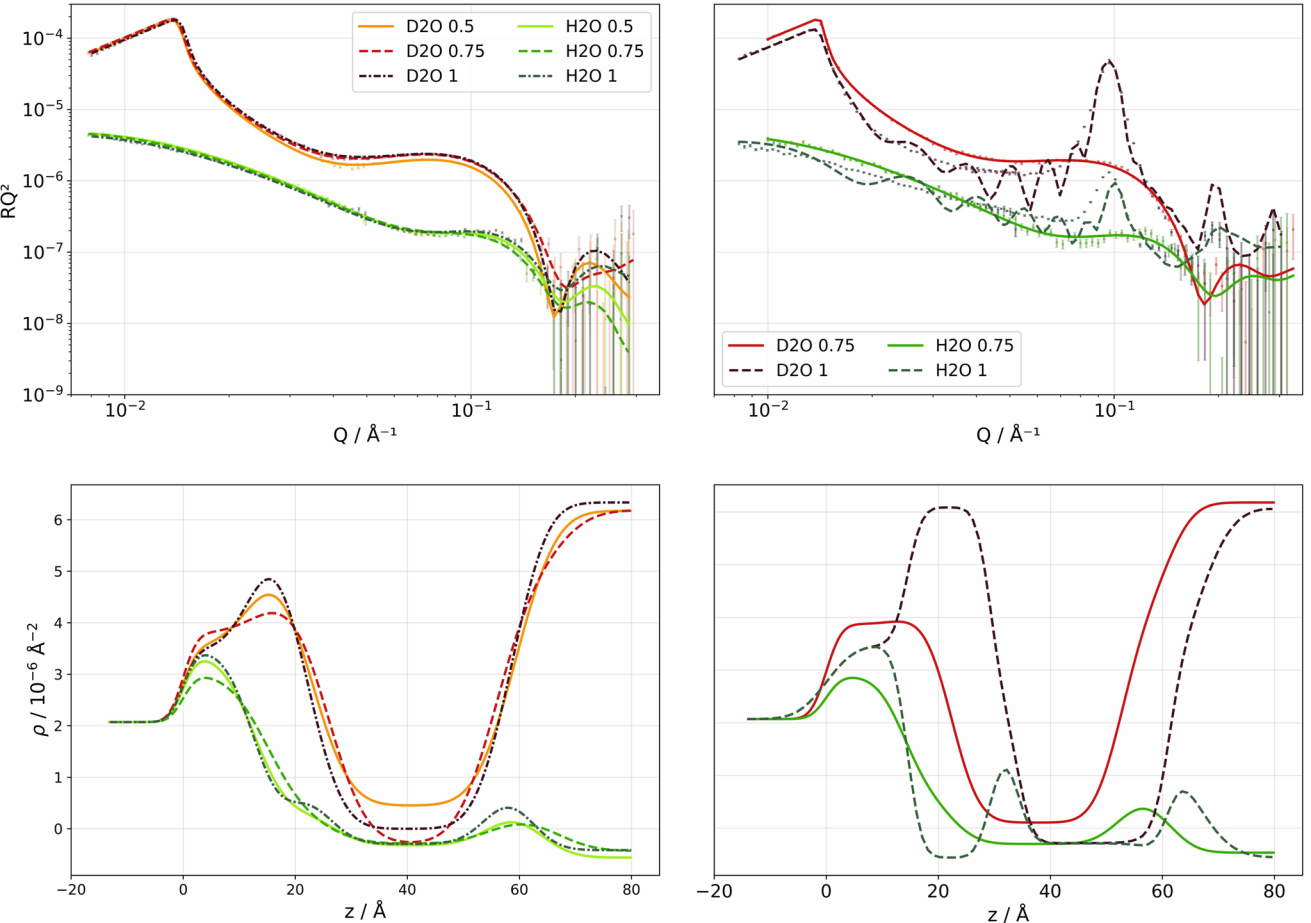
Neutron reflectometry analysis demonstrating concentration-dependent effects in h-POPC SALBs. The upper panels present reflectivity profiles for D_2_O and H_2_O contrasts with experimental data points and symmetrical bilayer model fits for (*a*) three lipid concentrations for IPA-based SALBs (0.5, 0.75 and 1.0 mg ml^−1^) and (*b*) EtOH-based SALBs (0.75 and 1.0 mg ml^−1^). The lower panels display the corresponding SLD profiles, revealing bilayer architecture evolution with concentration. The EtOH condition exhibits characteristic multilayer formation artefacts visible through extended oscillatory patterns in both reflectivity and SLD profiles, indicating surface aggregation beyond single bilayer deposition.

The establishment of optimal experimental conditions represents a critical prerequisite for systematic comparative investigation of SALB and VF methodologies. In a first step, the effect of lipid concentration and organic solvent selection on SLB formation efficiency was investigated using h-POPC as the model phospho­lipid system, with either IPA or EtOH as organic solvent (Table 1 ▸, Fig. 2 ▸). NR analysis using multiple isotopic contrast conditions is the most suitable method to perform a quantitative assessment of SLB structural parameters and surface coverage characteristics. Error bars on all fitted parameters were estimated using MCMC sampling (8000 iterations following 1000 burn-in steps) and represent the standard deviation of parameter values sampled around the best fit.

Fig. 2 ▸(*a*) gives the NR profiles obtained for an SLB formed by flowing either 0.5, 0.75 or 1.0 mg ml^−1^ h-POPC in IPA on solid–liquid cells and performing the solvent exchange using ultrapure water at a flow rate of 0.1 ml min^−1^. IPA was selected since previous investigations have demonstrated superior SLB formation efficiency for zwitterionic phospho­lipids in IPA compared with alternative organic solvents (Ferhan *et al.*, 2019 ▸; Tabaei, Choi *et al.*, 2014 ▸). NR data for 0.75 and 1.0 mg ml^−1^ h-POPC in EtOH are shown for comparative assessment in Fig. 2 ▸(*b*). The structural parameters of the IPA-based SALBs showed that the final SLB formed has reasonably well defined head thickness values ranging from 5.05 ± 0.08 Å to 8.2 ± 0.2 Å (0.5 and 1 mg ml^−1^ required a water layer in between SiO_2_ and the headgroup to obtain a good fit, while that was not the case for 0.75 mg ml^−1^, leading to the observed differences in headgroup thickness and coverages) and tail thicknesses varying from 27.7 ± 0.4 Å to 32.3 ± 0.2 Å regardless of the lipid concentration used when using IPA (Table 1 ▸).

The best data fits showed a systematic concentration-dependent coverage [Table 1 ▸, Fig. 2 ▸(*a*)]: surface coverage increased from 88.5 ± 0.1% at 0.5 mg ml^−1^ to 99.2 ± 0.9% at 0.75 mg ml^−1^, and plateaued there at 1.0 mg ml^−1^. For EtOH, however, the SLBs showed less satisfactory coverage, as expected from previous work (Ferhan *et al.*, 2019 ▸; Tabaei, Choi *et al.*, 2014 ▸). At 0.75 mg ml^−1^, the coverage was lower in EtOH than in IPA, while multilayer formation was observed for 1 mg ml^−1^ in EtOH, as indicated by a secondary fringe peaking at 10^−1^ Å^−1^ [Table 1 ▸, Fig. 2 ▸(*b*)]. This means that the optimal initial lipid composition occurs between 0.75 and 1 mg ml^−1^ for EtOH only, and also that the organic solvent choice has a significant impact on SLB quality. The generally observed lipid-concentration-dependent coverage progression aligns with established SALB formation mechanisms where insufficient lipid availability in the bulk reservoir limits complete surface coverage, while excessive lipid concentrations promote secondary nucleation events leading to multilayer formation or surface aggregates (Ferhan *et al.*, 2019 ▸). The phenomenological model developed by Gillissen *et al.* (2016 ▸) provides mechanistic insight into these concentration effects, establishing that the minimum lipid concentration required for complete bilayer formation (*c*_min_) depends on the boundary layer thickness (δ), bilayer mass density (ρ_LB_), flow rate (*Q*) and diffusion coefficient (*D*) according to
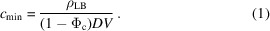
Here, Φ_c_ is the critical water fraction and *V* the volume of the cell. This relationship demonstrates that lipid concentration requirements are inherently system dependent, confirming previous results (Gillissen *et al.*, 2016 ▸).

### 3.2. Structural comparison as a function of SLB formation method: VF *versus* SALB

**Figure 3:**
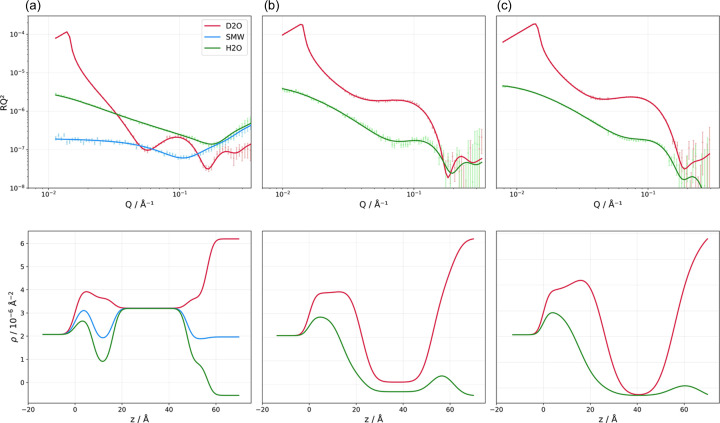
Neutron reflectometry profiles (symbols) and best fits (lines) for POPC SLBs formed via (*a*) calcium-mediated vesicle fusion, (*b*) solvent-assisted depletion in EtOH or (*c*) solvent-assisted depletion in IPA. (Top row) Methodology data, including measurements in D_2_O, SMW and H_2_O contrasts. (Bottom row) The corresponding SLD profiles for the best fits, revealing detailed bilayer architectures with characteristic head–tail–head organization. The different SLD values for the acyl tail region between VF SLBs and SALBs reflect the deuteration state differences in the lipids used: one tail deuterated in (d/h-) POPC for VF [data reproduced with permission from Åkesson *et al.* (2012 ▸), copyright (2012) RSC] and refitted *versus* h-POPC for SALBs. The temperature of SLB formation and characterization was 25°C.

Given that an alcohol is used in the formation of SLBs via the SALB method, it is worth performing a direct structural comparison between SLBs formed via VF [Fig. 3 ▸(*a*)] and SALB methods using NR [EtOH-based, Fig. 3 ▸(*b*), and IPA-based, Fig. 3 ▸(*c*)]. These results are presented in Table 2 ▸. VF-prepared SLBs exhibited a headgroup layer thickness of 8.6 ± 0.3 Å, demonstrating excellent agreement with EtOH- and IPA-based SALBs (8.6 ± 0.4 Å and 8.2 ± 0.2 Å, respectively). Table 2 ▸ shows that the acyl hydro­phobic core revealed a clear thinning across the methods as follows: VF (31.6 ± 0.4 Å) > EtOH-based SALB (30.1 ± 0.4 Å) > IPA-based SALB (27.7 ± 0.4 Å). The surface coverage of the acyl chain group is slightly better for IPA-based SALBs (99.2%) [Fig. 3 ▸(*c*)] than for EtOH-based SALBs (93.9%) [Fig. 3 ▸(*b*)].

The MMA for either headgroup or tail region was then calculated using the following formula:
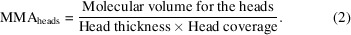
For the acyl tail layer (consisting of two leaflets), a multiplicative factor of 2 is added to this equation using the corresponding parameters for each tail. Ideally, the MMA values for the head and tail regions should be identical, which could be enforced using, for example, the *LipidLeafletGuest* function available in *RefnX*. However, throughout this paper (see *Methods* section), we chose not to impose any molecular constraints so as to have a systematic way of treating our SLBs regardless of coverage [SLBs with poor coverage present altered lipid packing geometry to decrease the exposure of the hydro­phobic core to the aqueous bulk solution (Jiang *et al.*, 2004 ▸)]. Note that the MMA values hereby obtained are consistent with previously reported values when experimental uncertainties are considered (Wacklin, 2011 ▸; Lind *et al.*, 2014 ▸; Vandoolaeghe *et al.*, 2009 ▸). We have calculated the discrepancy in MMAs across heads and tails, defined as the relative discrepancy of the tail towards the head and expressed as a percentage, and the results are presented in Tables 2 ▸ to 5. This analysis yields a discrepancy between the MMAs across headgroups and tails for all methods that may be statistically significant at times. This is comparable to the discrepancy reported for NR data on SLBs of low coverage, considering the larger errors reported in the early literature (Wacklin, 2011 ▸; Lind *et al.*, 2014 ▸; Vandoolaeghe *et al.*, 2009 ▸). Here, the discrepancy in MMA for the SALB method is twice as large as that for the VF method for SLBs of similar coverage, although without statistical significance. Despite the headgroup thicknesses being comparable between SALB- and VF-prepared SLBs, some alcohol could be present in the tail of these SLBs which could explain the observed differences in tail thickness as a function of the SLB formation method.

### 3.3. Determination of organic solvent retention in SALBs

**Figure 4:**
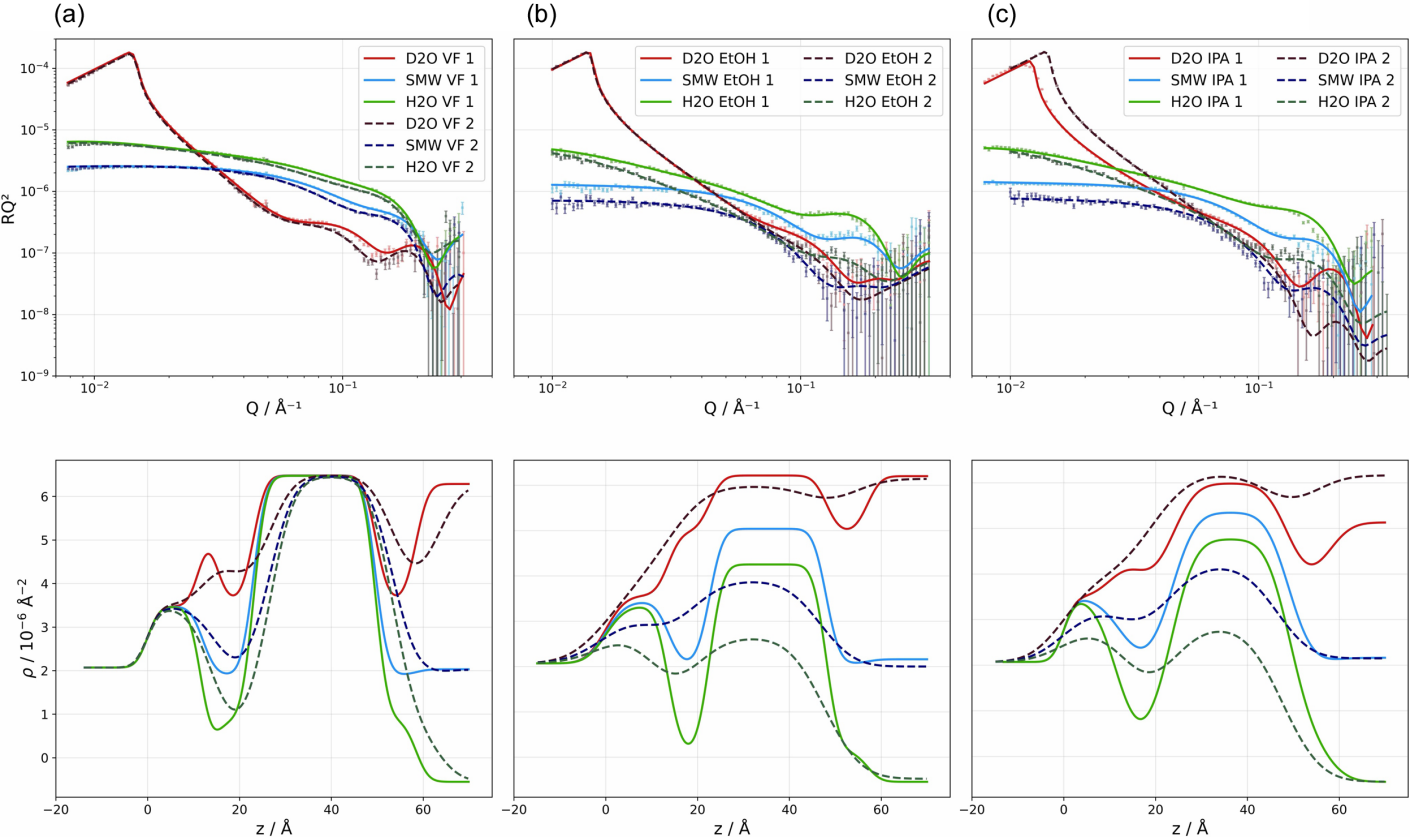
Neutron reflectometry analysis of d-DMPC bilayers, demonstrating organic solvent detection capabilities and reproducibility assessment. (Top row) Reflectivity profiles for (*a*) multiple VF replicates, and SALB formations using (*b*) EtOH and (*c*) IPA as solvents, with experimental data and *MixedSlab* model fits. (Bottom row) SLD profiles, showing detailed bilayer architectures with enhanced contrast from deuterated lipids. The profiles reveal the structural consistency between the VF and SALB methods, while enabling quantitative detection of organic solvent incorporation through modified SLD distributions in SALBs. Replicate measurements demonstrate the systematic variability in SALBs. The other two replicates for VF are given in Fig. S1. Both the EtOH-based measurement and the IPA-based SALB measurement 2 were performed at 25°C, the rest at 37°C. The solvent exchange was performed with ultrapure water.

NR in combination with selective deuteration can determine the presence of organic solvent remaining trapped within SALBs with a precision of a few vol.%. Here, we will use a different zwitterionic lipid in tail deuterated form, d-DMPC. This choice was made for two reasons: (i) DMPC is another common lipid used as a simple model for cellular membranes, thus enhancing the lipid portfolio used, and (ii) it is widely accessible in perdeuterated form for both tails and is considerably cheaper than POPC with the same degree of deuteration, though the latter can be secured via collaboration with deuteration facilities at large-scale facilities. Using DMPC allows verification of whether the high coverage achieved for POPC is attainable for other similar lipids without further optimization. Additionally, deuteration enhances the neutron scattering contrast between the tail region of the SLB and any residual hydrogenous organic solvent, owing to the greater difference in their SLD values compared with hydrogenated lipids. This enables the quantification of remaining solvent within the acyl chains in SLBs made by the SALB method (Table 3 ▸ and Fig. 4 ▸).

First, the baseline in the SLB structural variability was established by performing multiple replicates for the calcium-mediated VF approach for SLB deposition [Table 3 ▸, Fig. 4 ▸(*a*) and Fig. S1]. All VF-prepared SLBs were fitted with a water layer (2.0 to 2.9 Å) between the SLB and the underlying surface. The headgroup thickness ranged from 7.9 to 9.8 Å, while the acyl tail thickness ranged from 26.3 to 26.8 Å. All VF SLBs achieved complete surface coverage (99.5–99.9%), confirming calcium-mediated vesicle rupture reliability to form d-DMPC SLBs on silicon (Hardy *et al.*, 2013 ▸; Anderson *et al.*, 2009 ▸; Garcia-Manyes *et al.*, 2005 ▸).

EtOH-based SALBs [Fig. 4 ▸(*b*)] and IPA-based SALBs [Fig. 4 ▸(*c*)] presented structural parameters roughly consistent with the VF values with respect to headgroup and tail thickness values. However, significantly lower SLB coverage and reproducibility were found for the SALB method. The choice of solvent did not play a role in terms of coverage variability (21–30 percentage points between replicates), although the highest coverage was achieved with IPA as organic solvent [Table 3 ▸, Fig. 4 ▸(*c*)]. Low-coverage SLBs contain defects (Tabaei, Jackman, Kim, Yorulmaz *et al.*, 2015 ▸) which must imply a change in lipid packing in such a way that the headgroups spread along the defect edges to minimize the hydro­phobic core–water interface, leading to an increased headgroup area per molecule (Jiang *et al.*, 2004 ▸) and explaining the larger discrepancy in MMA between head and tails in agreement with literature values (Lind *et al.*, 2015 ▸). From the known organic solvent and acyl tail SLDs, as well as from fitting the tail region by assigning individual volume fractions for the acyl tail and organic solvent, we calculated the residual organic solvent in these low-coverage SALBs (Table 3 ▸, Fig. 4 ▸) to be up to 3.3 ± 0.9% for EtOH, while negligible alcohol amounts were detected for IPA. These results further position IPA as the preferable organic solvent for zwitterionic lipid SLB deposition, as previously suggested (Tabaei, Choi *et al.*, 2014 ▸). Assuming that no alcohol is present in the SLB, *i.e.* forcing the organic solvent content to be 0, the best fit to the data results in a tail thickness of 27.7 ± 0.3 Å, with comparable SLB coverage (46 vol.%) and slightly higher chi-squared values (Fig. S3). This demonstrates that the alcohol content can be determined accurately by NR. Note, though, that for those SLBs with higher coverage no significant level of alcohol is detected. Even if the headgroups are co-localized with the tail region, their total contribution to the SLD in that layer is insignificant in this case. For the lower-coverage SLB (<50 vol.%), alcohol is present, although we cannot disregard the possibility that some headgroups contribute to this value (Jiang *et al.*, 2004 ▸). To be able to confirm the degree of organic solvent incorporation, SALBs with h-lipid and d-organic solvent are needed, as seen in Section 3.4.

Tabaei, Choi *et al.* (2014 ▸) systematically tested organic solvents for DOPC SLB formation via the SALB method and found that IPA yields optimal results when rinsed with 10 m*M* Tris buffer with 150 m*M* NaCl at a flow rate of 50 µl min^−1^, characterized by the expected low-energy dissipation and the frequency shifts in QCM-D measurements, while EtOH and methanol produced intermediate results. *N*-Propanol showed higher dissipation values and decreased mobile fractions in fluorescence recovery after photobleaching studies, suggesting additional mass and viscoelastic elements in the bilayer. Our current results confirm IPA as the best organic solvent choice for zwitterionic lipids (Ferhan *et al.*, 2019 ▸), as higher average coverage is achieved when using IPA rather than EtOH for POPC. For d-DMPC replicates, IPA could yield a maximum coverage of 78%, while EtOH-based SALBs reached up to 71% coverage, using 0.75 mg ml^−1^ lipid. The relationship between flow rate and bilayer formation has been explained through a phenomenological model that considers the balance between micelle attachment and decomposition rates (Gillissen *et al.*, 2016 ▸). The model established that incomplete SLB formation occurs when flow rates are too high, resulting in insufficient lipid supply in the bulk reservoir, while excessively low flow rates can lead to suboptimal mixing and phase transition timing (Gillissen *et al.*, 2016 ▸).

The convergence of the current findings with previous systematic investigations supports a unified understanding of SALB reproducibility as an emergent property of multiple interacting parameters. The reproducibility challenges observed require simultaneous optimization of (i) sufficient lipid concentration to provide adequate nucleation sites, (ii) appropriate flow rates to allow effective micelle attachment and (iii) proper timing of the solvent exchange process to coincide with optimal lipid phase transitions (Ferhan *et al.*, 2019 ▸; Gillissen *et al.*, 2016 ▸). The current results demonstrate that achieving reproducible outcomes requires consideration of system-specific interactions determining optimal operating conditions, with coverage variability arising from the complex interplay between solvent properties, substrate interactions and kinetic formation processes. For 0.5 mg ml^−1^ d-DMPC, very low SLB coverage was obtained when the flow rate was set to 0.1 or 0.5 ml min^−1^. However, the slower flow rate yielded a higher coverage result (Fig. S3).

### 3.4. CaCl_2_-enhanced SALB formation and organic solvent detection in head and tail layers

**Figure 5:**
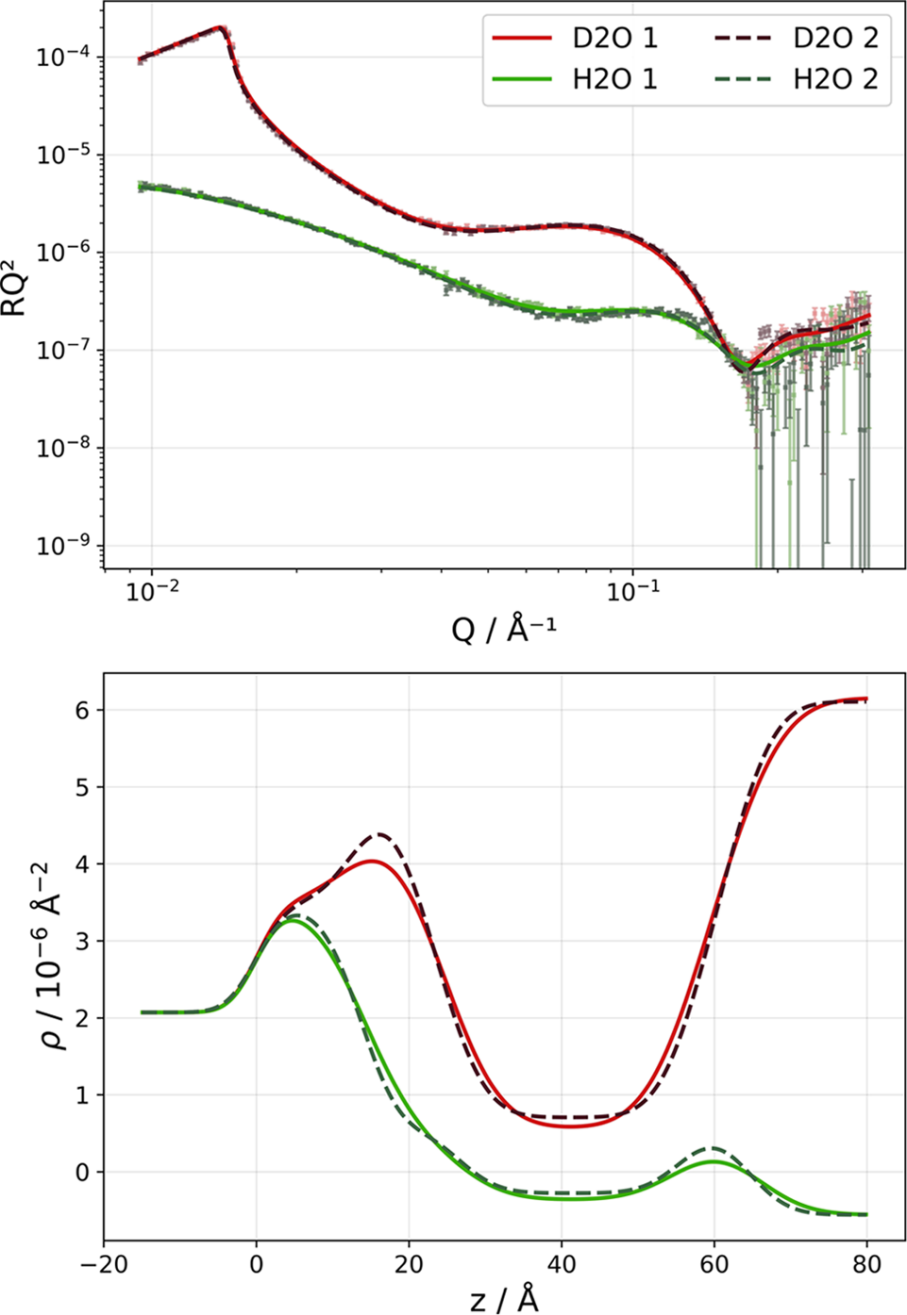
Neutron reflectometry analysis of CaCl_2_-enhanced h-DMPC/d-IPA SALB formation, demonstrating organic solvent detection capabilities in both head and tail bilayer regions. The upper panel displays reflectivity profiles for duplicate measurements, with experimental data and *MixedSlab* model fits demonstrating enhanced coverage and coverage reproducibility. The lower panel presents SLD profiles, revealing detailed bilayer architectures with quantitative organic solvent detection in both head and tail regions. The enhanced neutron contrast from the h-DMPC/d-IPA combination enables the determination of solvent incorporation throughout the bilayer structure. Calcium chloride mediated solvent exchange results in an improved formation reproducibility.

Given the low reproducibility and coverage in SALBs made so far, we attempted further protocol optimization. In this case, we used the established principles for the VF method in which calcium ions promote vesicle–substrate interactions in lipid membrane systems (Hardy *et al.*, 2013 ▸; Lind *et al.*, 2019 ▸; Chen *et al.*, 2018 ▸). Fig. 5 ▸ and Table 4 ▸ present the resulting NR data for two IPA-based SALBs formed in the presence of 1 m*M* CaCl_2_ at a flow rate of 0.1 ml min^−1^. In this case, d-IPA and h-DMPC were used to provide optimal neutron scattering contrast for quantitative detection of organic solvent incorporation within both headgroup and acyl tail regions of the SLBs, as the d-IPA has a significantly different SLD value from either tails or heads.

To start, the use of 1 m*M* CaCl_2_ during solvent exchange instead of ultrapure water resulted in substantial improvement in surface coverage and coverage reproducibility: 85.5 ± 0.8% *versus* 83.3 ± 0.6% (Table 4 ▸). This represents a significant advancement over IPA-based water-only protocols, as coverage was increased by 5–38 percentage points and the variability reduced from 30 percentage points (water rinse) to 2 percentage points (CaCl_2_ rinse).

The SLB structure formed with CaCl_2_ remained consistent with previous data for d-DMPC SLBs prepared via both VF and SALB. The acyl tail thickness values are constant (31.5 ± 0.4 Å and 31.9 ± 0.1 Å for SALB duplicates) and thicker than those from previous VF and ultrapure water SALB measurements (26.3–26.8 Å). These differences can be attributed to a transition from the fluid to the gel phase in the DMPC SLBs, which occurs at 24°C for h-DMPC (Chen *et al.*, 2018 ▸) and at around 19–20°C for d-DMPC (Wang & Chen, 1993 ▸; Guard-Friar *et al.*, 1985 ▸). The measurements for d-DMPC were made at 25 and 37°C, which give a fluid bilayer, while those for h-DMPC were made at room temperature (23°C). Thus, the h-DMPC SLB is expected to be in the ripple gel phase, which gives bilayers with thicker acyl chains than those in the fluid phase, as for d-DMPC (Prenner *et al.*, 1999 ▸). These values remain within the range of literature-reported values for phospho­lipid bilayers (Bagatolli & Sunil Kumar, 2009 ▸; Wadsäter *et al.*, 2013 ▸), confirming that calcium-mediated enhancement does not perturb fundamental SLB organization but does improve formation reliability. Further, previous studies showed that the extent of SLB coverage does not depend on the liquid crystalline phase upon vesicle fusion of DMPC (Lind *et al.*, 2014 ▸). Thus, it is unlikely that the increased coverage and reproducibility in 1 m*M* CaCl_2_ are due to the lipids being in the gel phase in this case.

On the basis of these results, the use of CaCl_2_ for the solvent exchange instead of ultrapure water keeps the remnant solvent below 2 vol.% in the acyl chain region, and negligible for the head region. This is consistent with the 3.3 ± 0.9% organic solvent within the acyl chain layer found when using ultrapure water for the solvent exchange. Since the coverage is significantly higher in these SLBs than for the data shown in Fig. 4 ▸, it is quite unlikely that the heads contribute significantly to the SLD of the tail region in this case.

The mechanism of CaCl_2_ enhancement probably involves charge bridging to enhance lipid–substrate interactions, analogous to mechanisms demonstrated in VF methodologies. Hardy *et al.* (2013 ▸) reported that divalent cations stabilize vesicle–substrate interactions between positively charged lipid headgroups and negatively charged substrates through charge bridging. Ca^2+^ ions contribute significantly to vesicle fusion even at concentrations as low as 25 µ*M*. The improved reproducibility with CaCl_2_ suggests that ionic interactions play a crucial role in SALB formation, similar to their importance in VF methods. Moreover, 1 m*M* CaCl_2_ is fully soluble in IPA, while this is not the case for higher salt concentrations. For instance, physiological salt concentrations cannot be flushed into a 20 vol.% EtOH solution in size-exclusion chromatography columns due to the formation of salt crystals in the columns. Therefore, chromatographic protocols always include a wash with ultrapure water in between 20% alcohol and buffers to avoid damaging the column. The presence of organic solvent in lipid bilayers has well documented effects on membrane properties: molecular dynamics simulations have demonstrated that equilibrium partitioning of EtOH into POPC bilayers (achieving approximately 8.5 vol.%) increases membrane fluidity and permeability through hydrogen bonding with lipid ester oxygen atoms at the head–tail interface (Patra *et al.*, 2006 ▸). NR analysis of the SALB systems shows that most of the residual alcohol coexists with the tail region. This distribution pattern is consistent with alcohol molecules binding at the head–tail interface via hydrogen bonding to ester oxygen atoms, while their hydro­phobic alkyl chains extend into the lipid hydro­carbon tails. Such positioning would contribute alcohol mass to the tail region as detected by NR, while simultaneously affecting the molecular volumes of both head and tail regions through interfacial binding interactions. This mechanism could explain the larger variation in MMA values observed across different bilayer regions. Note that implementing an asymmetric model in which the inner and outer headgroup regions are fitted independently did not improve the quality of the fit or the differences across MMA values.

The use of 1 m*M* CaCl_2_ during the SALB process represents a significant advancement in the reliability of SLB formation, achieving coverage reproducibility comparable to that of VF methodologies while maintaining the expanded compositional and substrate compatibility advantages of solvent exchange approaches (we are currently working on bacterial model membranes using this approach). The improved reproducibility observed in h-DMPC systems reflects the effects of calcium-mediated stabilization compared with previous d-DMPC investigations. Organic solvent incorporation showed values within the range of the ultrapure-water-rinsed SALBs, establishing reference concentration ranges for residual organic solvent that should be considered when interpreting results from protein–membrane interaction studies using these model membrane systems. While substantial improvement in coverage reproducibility was observed, complete substrate coverage may be achieved by increasing the lipid concentration or reducing the temperature, as lipid coverage is also temperature dependent (Hohner *et al.*, 2010 ▸). Finally, the enhanced contrast conditions enabled, to the best knowledge of the authors, the first direct measurement of organic solvent distribution between the head and tail bilayer regions, while systematically evaluating the efficacy of calcium-mediated improvements to SALB formation protocols (Table 4 ▸, Fig. 5 ▸).

### 3.5. Suitability of the use of SALBs for intermolecular binding studies

SLBs are often used as model cell membranes to investigate, for example, membrane–peptide interactions. Given the residual presence of alcohol and the observed effects in acyl tail thickness, as well as the MMA inconsistency across headgroup regions and tails, it is worth providing a critical validation for their use compared with VF methodologies. Here, the well studied antimicrobial peptide melittin (5 µ*M*) was used to follow the interaction with d-DMPC SLBs prepared via the SALB method (in this particular case, ultrapure water was used for the solvent exchange). Melittin is a 26 amino acid cationic antimicrobial peptide (+6 charge at physiological pH) from honeybee venom. It serves as an ideal model system for comparative peptide–membrane interaction analysis due to its well characterized disruption mechanisms and extensive literature documentation (Clifton *et al.*, 2020 ▸; Krueger *et al.*, 2001 ▸).

**Figure 6:**
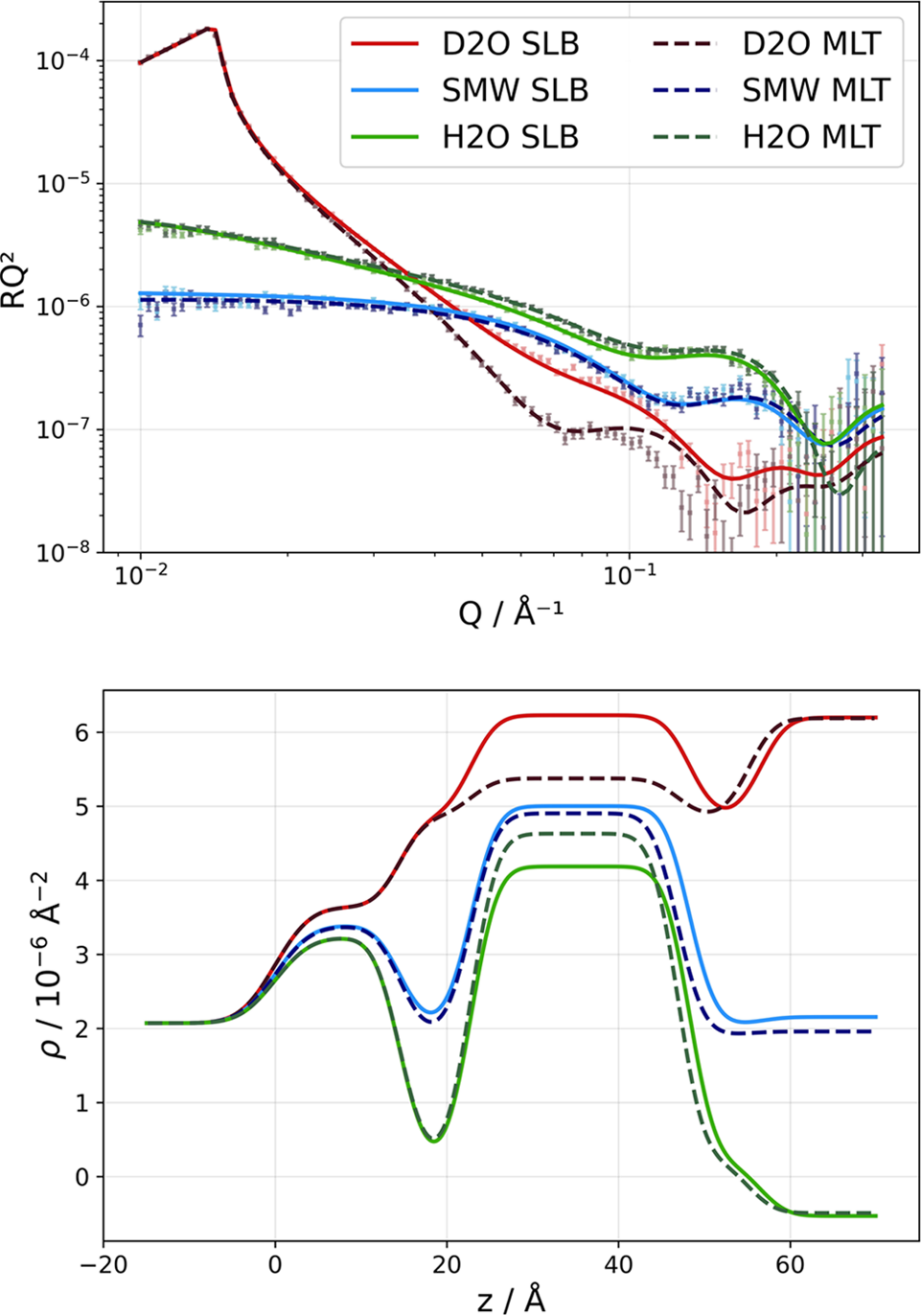
Neutron reflectometry analysis of melittin (MLT) interactions of a low-coverage (69.7 ± 0.8%) SALB bilayer. The upper panel displays reflectivity profiles for D_2_O, H_2_O and SMW contrasts before and after 5 µ*M* melittin incubation, with experimental data points and *MixedSlab* model fits. The lower panel presents SLD profiles, revealing the melittin interaction. The low-coverage SALB bilayer maintains structural integrity despite enhanced peptide incorporation, due to melittin binding into existing defects and holes.

The addition of melittin to high-coverage SLBs is known to induce a characteristic membrane disruption that involves lipid removal, shown by an SLB coverage reduction, and lipid bilayer tail core thinning (Clifton *et al.*, 2020 ▸). The thinning is typically accompanied by melittin incorporation into the SLB tail core. For example, 18 vol.% melittin was found in an SLB made of an 8:2 molar ratio DMPC:DMPG mixture (Clifton *et al.*, 2020 ▸). Since DMPC is less charged than DMPG, it is expected that melittin will bind less strongly to our neutral d-DMPC bilayers (Clifton *et al.*, 2020 ▸; Pan & Khadka, 2016 ▸; Wessman *et al.*, 2008 ▸). In fact, we found that the addition of melittin to the EtOH-based DMPC SALB 1 sample (the pristine SALB was fitted as pure lipid for simplicity, as it originally contained negligible amounts of alcohol; Table 2 ▸) did not change the initial SLB coverage (71 ± 5% after melittin incorporation instead of the initial 69.7 ± 0.8%) but led to the expected small acyl chain thinning (from 25.4 to 24.1 Å) and was accompanied by 18 ± 5 vol.% peptide incorporation into the acyl chain layer (Table 5 ▸, Fig. 6 ▸). The incorporation of unexpectedly high amounts of peptide for the zwitterionic lipid aligns well with the expected interaction pattern in low-coverage bilayers. Atomic force microscopy (Tabaei, Guo *et al.*, 2016 ▸) studies demonstrated that melittin preferentially accumulates at bilayer defects, with the defects serving as nucleation sites for peptide binding. Defects expose the underlying negatively charged surface and, given they are large enough, can accommodate significant peptide loads without requiring the classical membrane disruption mechanisms observed in intact bilayers (Tabaei, Guo *et al.*, 2016 ▸). The 18 vol.% melittin incorporation observed in our low-coverage SALB is thus likely to be due to the anionic SiO_2_ substrates exerting attractive electrostatic forces on the cationic melittin through bilayer defects.

In summary, the observed melittin interactions proceeded as expected from literature mechanisms despite possible residual organic solvent incorporation, indicating that alcohol concentrations at these levels do not significantly disrupt melittin–membrane interactions. However, the persistent presence of organic solvent in the acyl chain layer in SALBs necessitates careful consideration when extending this validation to other protein systems. Melittin is incorporated directly within the lipid tail region (Clifton *et al.*, 2020 ▸) where residual alcohol is most concentrated, yet exhibits literature-consistent interaction mechanisms. This tolerance may be specific to melittin’s interaction mechanism and structural characteristics.

The broader implications of organic solvent presence on protein–membrane interactions must be considered within established protein stability principles. Griebenow & Klibanov (1996 ▸) systematically investigated protein denaturation across aqueous–organic mixtures using aceto­nitrile, propan-1-ol and tetra­hydro­furan, demonstrating that proteins are more de­natured in these mixtures than in pure organic solvents due to kinetic control effects. Their detailed analysis of lysozyme in aceto­nitrile–water mixtures showed that, even at relatively low concentrations of 10 vol.% aceto­nitrile, the α-helix content decreased by approximately 2 percentage points (from 34 to 32 vol.%) compared with the case of pure water. More pronounced denaturation occurred at higher concentrations, particularly around 60 vol.% where the α-helix content dropped by 21–23 percentage points (from 34% to 11–13%) (Griebenow & Klibanov, 1996 ▸). Mechanistically, this occurs because, in aqueous–organic mixtures, proteins retain sufficient molecular mobility for denaturation to occur, while in pure organic solvents, proteins are kinetically trapped in their native-like conformations due to restricted conformational mobility (Griebenow & Klibanov, 1996 ▸; Rupley & Careri, 1991 ▸). However, these effects might be minimized in SALBs since the acyl region where the alcohol resides is hydro­phobic in nature.

## 4. Conclusions

This work provides a systematic comparison of the SALB method with the well established VF method in terms of SLB structure and coverage using well characterized phospho­lipids. For the SALB method, SLB coverage was found to be affected by lipid concentration, selection of organic solvent and temperature. Thus, the methodological conditions should be optimized for each desired lipid composition. Improvements in coverage and reproducibility were consistently seen when using IPA instead of EtOH, and when the washing step was performed with 1 m*M* CaCl_2_ instead of ultrapure water. Our results indicate that, besides the choice of alcohol, the lipid concentration is the main factor that should be varied in order to achieve a high-coverage SLB.

A key concern with the SALB method is retention of the alcohol after solvent exchange and how it might affect protein–membrane interactions. Here, using contrast variation available through NR with selective deuteration, we quantified that the alcohol retention reaches at most 3.3% in the tails, which could explain differences in the SLB core thickness compared with SLBs made via VF for both lipids studied. The presence of alcohol in the headgroup region is negligible.

Preliminary tests with the well known antimicrobial peptide melittin showed that peptide–membrane interactions followed trends observed previously on SLBs produced by VF. While this is likely to be protein dependent, these results show that the SALB method can be utilized to study protein–membrane interactions on SLBs not achievable through VF methods.

## Supplementary Material

Supporting information file. DOI: 10.1107/S1600576726000312/roo5006sup1.pdf

## Figures and Tables

**Table 1:** Systematic analysis of h-POPC bilayer formation efficiency across lipid concentrations using IPA (0.5, 0.75 and 1.0 mg ml^−1^) and EtOH (0.75 and 1.0 mg ml^−1^) as organic solvents Data include head and tail layer thickness measurements (Å) with statistical uncertainties, surface coverage percentages derived from neutron reflectometry modelling, and structural parameters obtained through symmetrical bilayer fitting procedures. The concentration series demonstrates systematic trends in bilayer formation efficiency, with optimal conditions established for subsequent comparative investigations. All measurements were performed using the standard SALB protocol with 0.1 ml min^−1^ flow rate and 70 min solvent exchange duration.

	Layer	Thickness (Å)	Coverage (%)	MMA (Å^2^)
IPA-based SALB, 0.5 mg ml^−1^	Head	5.04 ± 0.04	66 ± 3	100 ± 3†
	Tail	32.3 ± 0.2	88.5 ± 0.1	64.6 ± 0.4†
IPA-based SALB, 0.75 mg ml^−1^	Head	8.2 ± 0.2	50 ± 2	80 ± 4‡
	Tail	27.7 ± 0.4	99.2 ± 0.9	67 ± 1‡
IPA-based SALB, 1 mg ml^−1^	Head	5.04 ± 0.05	80 ± 2	82 ± 2†
	Tail	31.1 ± 0.2	95.6 ± 0.4	62.2 ± 0.4†
EtOH-based SALB, 0.75 mg ml^−1^	Head	8.6 ± 0.3	49 ± 2	80 ± 5‡
	Tail	30.1 ± 0.4	93.9 ± 0.7	65 ± 1‡
EtOH-based SALB, 1 mg ml^−1^	Head bilayer	5.01 ± 0.01	90 ± 4	73 ± 3
	Tail bilayer	26.6 ± 0.2	99.9 ± 0.3	69.6 ± 0.6
	Multilayer solvent (×6)	27.14 ± 0.06		
	SLB (×6)	38.00 ± 0.05	89.1 ± 0.1	

† These SLBs showed statistically significant differences in MMA between head and tails at the 99.99% confidence level.

‡ These SLBs showed statistically significant differences in MMA between head and tails at the 99% confidence level.

**Table 2:** Comparative structural analysis of h-POPC bilayers formed via VF and SALB methodologies using EtOH and IPA organic solvents Parameters include head and tail region thickness measurements (Å) with statistical uncertainties and surface coverage percentages derived from neutron reflectometry modelling. The data demonstrate a quantitative structural comparability between formation methods while revealing systematic coverage differences characteristic of SALBs. The analysis employed symmetrical bilayer models with SiO_2_ substrate interaction corrections and appropriate contrast matching protocols. The structural parameters for the underlying surface and roughness parameters are given in Table S2. The SALB data were obtained from h-POPC systems as described in the *Methods* section, while VF data were derived from refitted d/h-POPC bilayers published by our team earlier (Åkesson *et al.*, 2012 ▸). Error propagation was used for the calculation of MMA and the discrepancy in MMA.

	Layer	Thickness (Å)	SLB coverage (vol.%)	MMA (Å^2^)	Discrepancy in MMA (%)
VF	Head	8.6 ± 0.3	55 ± 2	66 ± 3	11 ± 5†
	Tail	31.6 ± 0.4	99.8 ± 0.2	59.2 ± 0.7	
EtOH-based SALB, 0.75 mg ml^−1^	Head	8.6 ± 0.3	49 ± 2	80 ± 5	23 ± 8‡
	Tail	30.1 ± 0.4	93.9 ± 0.7	65 ± 1	
IPA-based SALB, 0.75 mg ml^−1^	Head	8.2 ± 0.2	50 ± 2	80 ± 4	19 ± 6‡
	Tail	27.7 ± 0.4	99.2 ± 0.9	67 ± 1	

† These SLBs showed statistically significant differences in MMA between head and tails at the 95% confidence level.

‡ These SLBs showed statistically significant differences in MMA between head and tails at the 99% confidence level.

**Table 3:** Structural parameters and compositional analysis of d-DMPC SLBs comparing VF and SALB methodologies, enabling quantitative organic solvent detection The dataset includes multiple VF replicates (samples VF 1 to VF 4), and SALBs using EtOH and IPA each in duplicate. The data demonstrate a structural consistency between the formation methods, while revealing systematic organic solvent incorporation up to a level of 4 ± 1 vol.% in SALBs. The structural parameters for the underlying surface and roughness parameters are given in Table S2. The solvent exchange was performed with ultrapure water.

	Layer	Thickness (Å)	Coverage (%)	Organic solvent (%)	MMA (Å^2^)	Discrepancy in MMA (vol.%)
VF 1, 37°C	Head	8.80 ± 0.08	59.7 ± 0.5		63.1 ± 0.8	7 ± 1†
	Tail	26.59 ± 0.04	99.9 ± 0.1		58.9 ± 0.1	
VF 2, 37°C	Head	9.8 ± 0.2	54.2 ± 0.9		62 ± 2	6 ± 3
	Tail	26.8 ± 0.1	99.5 ± 0.5		58.7 ± 0.4	
VF 3, 37°C	Head	8.95 ± 0.08	60.7 ± 0.5		60.9 ± 0.7	2 ± 1‡
	Tail	26.3 ± 0.04	99.94 ± 0.07		59.5 ± 0.1	
VF 4, 37°C	Head	7.85 ± 0.1	66.8 ± 0.7		63 ± 1	8 ± 2†
	Tail	26.73 ± 0.05	99.9 ± 0.2		58.5 ± 0.1	
EtOH-based SALB 1, 25°C	Head	9.0 ± 0.3	28 ± 1		132 ± 6	52 ± 7§
	Tail	25.4 ± 0.3	70.7 ± 0.9	0.2 ± 0.2	87 ± 2	
EtOH-based SALB 2, 25°C	Head	5.1 ± 0.1	24 ± 2		264 ± 30	130 ± 26†
	Tail	29.6 ± 0.4	49.5 ± 0.5	3.3 ± 0.9	115 ± 2	
IPA-based SALB 1, 37°C	Head	8.0 ± 0.6	46 ± 4		89 ± 10	14 ± 13
	Tail	25.5 ± 0.5	78 ± 1	0.3 ± 0.5	78 ± 2	
IPA-based SALB 2, 25°C	Head	5.04 ± 0.05	26 ± 2		252 ± 20	105 ± 16§
	Tail	26.8 ± 0.4	47.6 ± 0.6	0.8 ± 0.4	123 ± 2	

† These SLBs showed statistically significant differences in MMA between head and tails at the 99.9% confidence level.

‡ These SLBs showed statistically significant differences in MMA between head and tails at the 95% confidence level.

§ These SLBs showed statistically significant differences in MMA between head and tails at the 99.99% confidence level.

**Table 4:** Enhanced SALB formation analysis using 1 m*M* CaCl_2_-mediated solvent exchange with h-DMPC/d-IPA systems Parameters include head and tail thickness measurements (Å) with statistical uncertainties, surface coverage percentages, and organic solvent volume fractions detected within both head and tail regions through contrast enhancement. The data demonstrate a systematic improvement in coverage reproducibility and enable a quantitative assessment of organic solvent incorporation across the complete bilayer architecture. The analysis employed *MixedSlab* modelling with independent determination of lipid and alcohol volume fractions in both head and tail regions. Duplicate measurements confirm increased coverage and enhanced protocol reproducibility compared with water-only solvent exchange procedures. The structural parameters for the underlying surface and roughness parameters are given in Table S2. Measurements were done at room temperature.

	Layer	Thickness (Å)	Coverage (vol.%)	Organic solvent (vol.%)	MMA (Å^2^)	Discrepancy in MMA (%)
d-IPA, replicate 1	Head	7.3 ± 0.6	54 ± 6	0.4 ± 0.5	84 ± 10	47 ± 18†
	Tail	31.5 ± 0.4	85.5 ± 0.8	0.6 ± 0.5	57.0 ± 0.8	
d-IPA, replicate 2	Head	6.2 ± 0.2	65 ± 2	0.2 ± 0.3	83 ± 4	43 ± 7‡
	Tail	31.9 ± 0.1	83.3 ± 0.6	2.0 ± 0.5	57.9 ± 0.3	

† These SLBs showed statistically significant differences in MMA between head and tails at the 99.9% confidence level.

‡ These SLBs showed statistically significant differences in MMA between head and tails at the 99.99% confidence level.

**Table 5:** Comparative analysis of melittin interactions with high-coverage VF bilayers and low-coverage SALB bilayers using d-DMPC systems Parameters include structural measurements before and after 5 µ*M* melittin incubation, with head and tail thickness values (Å) and surface coverage percentages. The data demonstrate coverage-dependent interaction mechanisms, with high-coverage systems exhibiting classical membrane disruption (lipid removal, bilayer thinning) while low-coverage systems show enhanced peptide incorporation without structural perturbation. Reference model analysis confirms that the apparent head thickness changes in SALB systems arise from bilayer asymmetry rather than melittin-induced modifications. The structural parameters for the underlying surface and roughness parameters are given in Table S2. For simplicity the SALB – prior to and after melittin incubation – was fitted without the negligible 0.2 ± 0.2% EtOH component in the acyl tail region.

	Prior to melittin	After melittin
Head thickness (Å)	8.3 ± 0.2†	8.3 ± 0.2†
Tail thickness (Å)	25.4 ± 0.3	24.1 ± 0.3
Head coverage (vol.%)	30.4 ± 0.8†	30.4 ± 0.8†
Tail coverage (vol.%)	69.7 ± 0.8	71 ± 5
Head MMA	131 ± 5	131 ± 5
Tail MMA	88 ± 1	92 ± 30
Discrepancy in MMA	49 ± 6†	42 ± 36

† The head thickness and head volume fraction were co-fitted between the SLB before and after melittin insertion.

‡ These SLBs showed statistically significant differences in MMA between head and tails at the 99.99% confidence level.
