# Supplementary material for: Structure and composition of supported lipid bilayers: a comparison between solvent-assisted lipid bilayer and vesicle fusion methods
Source: J Appl Crystallogr. 2026 Feb 27;59(Pt 2):343–56. doi: 10.1107/S1600576726000312 (PMC13060456; doi:10.1107/S1600576726000312)
Supplement: Supplementary file 1 [file j-59-00343-sup1.pdf]

JOURNAL OF APPLIED CRYSTALLOGRAPHY

Volume 59 (2026)

Supporting information for article:

Structure and composition of supported lipid bilayers: a comparison between solvent-assisted lipid bilayer and vesicle fusion methods

Birgit Felderer, Claire Buchanan, Shuhong Wang, Hsin-Hui Shen, Andrew Nelson, Stephen C. L. Hall and Marité Cárdenas

Methodology, NR data analysis

In all cases, the thickness, volume fraction (1 - coverage) and roughness were fitting parameter. Whereas the roughness of the SiO_2 was fitted individually and all other layers (solvent layer to outer heads) were cofitted. The MMA for each head and tail layer was calculated and the MMA error was calculated via an error propagation. The MMA error of the tails is calculated similar to the one of the heads, but divided by two to account for the two acryl tail layers.

MMA error heads = $\sqrt{(\text{vfsolv\_head\_err}/\text{lipid\_coverage\_head})^2 + (\text{t\_head\_err}/\text{t\_head\_val})^2)}$

The SLD of the d-DMPC was fitted for the SLBs formed via the VF. This average value was used for d-DMPC IPA-based SALB 1 (SLD = 6.49), while d-DMPC IPA-based SALB 2 as well as d-DMPC EtOH-based SALB 1 and 2 were fitted with an SLD of 6.24, as a different lipid batch was used in this case. The corresponding SLD value was fitted from a VF bilayer performed with that lipid batch (Data not shown). Ranges for fitting parameters are found in SI Table 4. The reason behind the variability in SLD across batches depends on different level of deuteration across d-DMPC batches and the presence of a range of species with different extent of deuteration in each batch.

Table S1 shows the SLD values used as well as the volumes used for the MMA calculation.

Table S1: SLD values and Volume/molecule

Layer	***SLD***	***Volume (Å³)***
Si	*2.07*	
SiO_2	*3.47*	
h/d-POPC Head<br>*Tail*	*1.81*<br>*3.2*	*331*<br>*933.7*
h-POPC Head<br>*Tail*	*1.81*<br>*-0.29*	*331*<br>*925*
h-DMPC Head<br>*Tail*	*1.81*<br>*-0.379*	*331*<br>*769*
d-DMPC Head<br>*Tail*	*1.81*<br>*6.24 / 6.49*	*331*<br>*782*
h-ISO	*-0.327*	
d-ISO	*6.179*	
h-EtOH	*-0.344*	
Melittin	*1.444*	

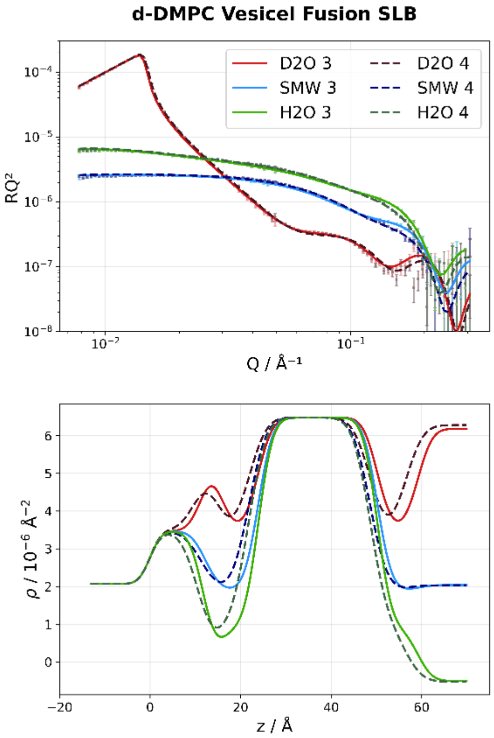

Figure S1: Neutron reflectometry analysis of VF d-DMPC bilayer 3 and 4. Upper panels display reflectivity profiles (RQ^2 vs Q). Lower panels present scattering length density (SLD) profiles ($\rho/10^{-6}$ Å^{-2} vs z/Å). The temperature of SLB formation and characterization was 37°C.

Sample	*SiO2*			*Solvent*	*SLB*
	Thickness (Å)	*Coverage (%)*	*Roughness (Å)*	*Thickness (Å)*	*Linked Roughness*
VF h/d-POPC; 25°C	7.6 ± 0.3	82 ± 2	2.2 ± 0.02	/	2.1 ± 0.1
0.5 mg/mL IPA h-POPC; 25°C	13.6 ± 0.2	97.9 ± 0.6	2.05 ± 0.06	5.9 ± 0.1	4.99 ± 0.02
0.75 mg/mL IPA h-POPC; 25°C	18.8 ± 0.4	80.3 ± 0.5	2.7 ± 0.6	/	5.0 ± 0.2
0.75 mg/mL EtOH h-POPC; 25°C	13.8 ± 0.2	85.1 ± 0.8	2.2 ± 0.2	/	3.9 ± 0.3
1 mg/mL IPA h-POPC; 25°C	13.1 ± 0.1	99.9 ± 0.1	2.03 ± 0.05	6.46 ± 0.08	4.0 ± 0.1
1 mg/mL EtOH h-POPC; 25°C	14.6 ± 0.2	99.98 ± 0.02	2.02 ± 0.02	14.9 ± 0.1	4.3 ± 0.1
VF d-DMPC 1; 37°C	11.92 ± 0.05	99.9 ± 0.2	2.01 ± 0.01	2.36 ± 0.08	2.16 ± 0.07
VF d-DMPC 2; 37°C	14.5 ± 0.1	99 ± 0.6	2.3 ± 0.2	2.3 ± 0.2	4.2 ± 0.1
VF d-DMPC 3; 37°C	12.09 ± 0.05	99.94 ± 0.07	2.01 ± 0.01	2.89 ± 0.07	2.53 ± 0.07
VF d-DMPC 4; 37°C	11.1 ± 0.2	99.3 ± 0.7	2.01 ± 0.02	2.9 ± 0.1	3.05 ± 0.07
0.75 mg/mL EtOH d-DMPC 1; 25°C	13.6 ± 0.3	96 ± 1	3.4 ± 0.4	/	2.3 ± 0.4
0.75 mg/mL EtOH d-DMPC 2; 25°C	12.1 ± 0.4	85 ± 1	6.6 ± 0.2	/	5.93 ± 0.08
0.75 mg/mL IPA d-DMPC 1; 37°C	12.05 ± 0.06	99.91 ± 0.08	2.02 ± 0.02	3.5 ± 0.5	4.1 ± 0.3
0.75 mg/mL IPA d-DMPC 2; 25°C	15.9 ± 0.2	84.0 ± 0.6	5.96 ± 0.05	/	5.97 ± 0.04
0.75 mg/mL d-IPA h-DMPC 1; Room temperature	14.9 ± 0.1	99.8 ± 0.3	2.9 ± 0.2	3.1 ± 0.6	5.6 ± 0.2
0.75 mg/mL d-IPA h-DMPC 2; Room temperature	14.66 ± 0.09	99.8 ± 0.3	3.1 ± 0.1	4.5 ± 0.2	4.5 ± 0.1
0.75 mg/mL EtOH d-DMPC 1 + Melittin, 25°C	14.5 ± 0.2	93.9 ± 0.8	3.2 ± 0.3	/	2.4 ± 0.3

Table S2: Structural parameters for the underlying surface and roughness parameters of all analysed SLDs.
**Linked roughness: between solvent and bilayer*

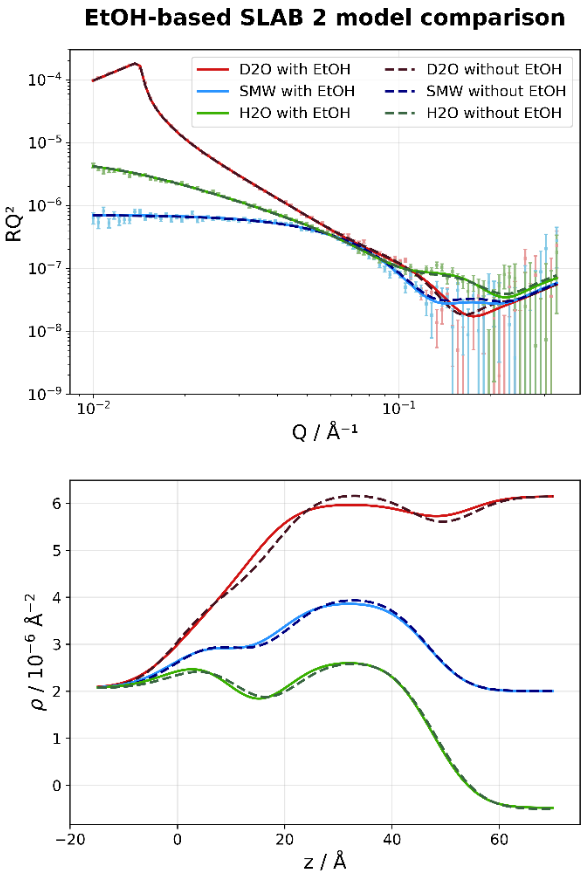

Figure S2 EtOH-based SALB 2 fitted with (MixedSlab) to enable the determination of alcohol in the SLB versus a simple Slab fit without any organic solvent. The total Chi² values rise from 1276 (2 SiO_2 and 3 SLB contrasts) to 1312 when fitting the SLB without organic solvent component.

The following ranges for fitted values were used

Table S3: Bounds used for fitting

Layer	Thickness (Å)	Volume Fraction	Roughness (Å)
SiO2	5 – 25	0.0 – 0.3	2 - 6/8
Solvent	0 – 10	Fixed to 1	2 – 5/6
Inner Heads	6 – 10, 5-12	0 – 0.7/ 0.8/ 1	2 – 5/6
Acryl Tails	24 – 32/35, 22 – 32	0 – 0.5	2 – 5/6
Outer Heads	6 – 10	0 – 0.7/ 0.8/ 1	2 – 5/6
Mixed Slab: Organic Solvent		0.0 – 0.1	
Mixed Slab: Melittin		0 – 0.5	
Mixed Slab: Lipid		0.3 – 0.7 or 0.5 – 1	

For all samples that are not specifically named below the first bound was used (alias 24-32 Å for the acryl tails thickness).
The head thickness from 5-12 Å was used for the IPA-based POPC SALB (0.5 and 0.75 mg/mL), the EtOH-based POPC SALB (1 mg/mL), IPA-based dDMPC SALB 1 and 2 and EtOH-based dDMPC 1 and 2
The tail thickness up to 35 Å was used for the VF POPC and IPA-based POPC SALB with 0.5 mg/mL, and 22-32 Å was exclusively used for the Melittin interaction study to allow for tail thinning. A volume fraction of 0-0.8 was used for EtOH-based d-DMPC SALB 1 and the melittin interaction study, and 0-1 was used for EtOH-based d-DMPC SALB EtOH 2. Regarding the Bound of the organic solvent: This is the ratio of organic solvent in lipid that afterwards gets normalized in perspective of the solvent in the layer. The SiO_2 roughness is fitted as 2-8 Å for the EtOH-based SALB 2, and a SLB roughness of 2-5 Å for the IPA-based POPC SALB 0.5 mg/mL. The multilayer of EtOH-based POPC SALB (1 mg/mL) had as the solvent layer restriction of 0-30 Å, 30-53 Å for the multilayer, 0.1-1, 1-30 for the volume fraction, and 0-5 Å for the roughness.

As preliminary experiments, we measured angle 1 (H_2O) for 2 SLBs after the solvent exchange:
IPA-based SALB: 0.5 mg/mL dDMPC - rinse: 30 min 0.5 ml/min ultrapure water and 0.5 mg/mL dDMPC - rinse 60 min 0.1 mL/min ultrapure water.
In both cases there was not enough SLB deposition, so the second angle was not collected, nor a different contrast. Hence, using these data we can only state that the SLB coverage was very low, as depicted in SI Figure 2. However, it can be seen that the measurement of the SLB obtained by the slower flowrate (0.1 mL/min, Sample, SI Figure 2 right) is more similar to the IPA-based SALB 1 than the SLB measurement after using a higher flow rate (0.5 ml/min, left).

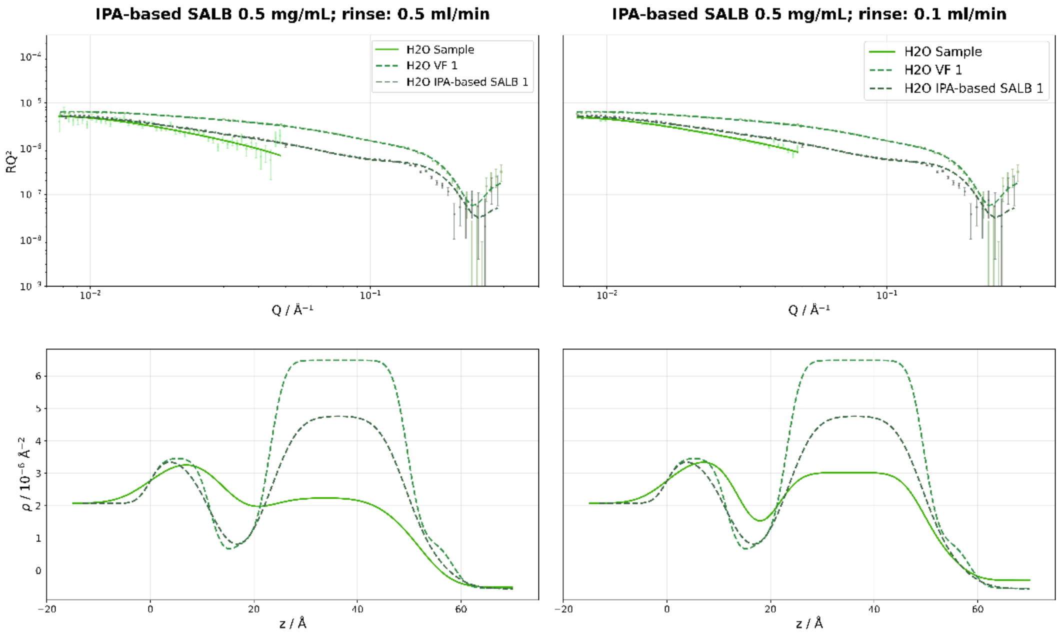

Figure S3. First angle of H_20 dDMPC 0.5 mg/mL IPA-based SALB- rinse: 30 min 0.5 ml/min ultrapure water (Sample left) and first angle of H_20 dDMPC 0.5 mg/mL IPA-based SALB- rinse: 60 min 0.1 ml/min ultrapure water (Sample right). H_2O of the high coverage VF SLB 1 (99.9 % coverage) and medium coverage IPA-based SALB 1 (79 % coverage) are depicted as comparison.
